# Inhibition of intimal hyperplasia in murine aortic allografts by administration of a small-molecule TLR4 inhibitor TAK-242

**DOI:** 10.1038/s41598-017-16160-4

**Published:** 2017-11-17

**Authors:** Chuangyan Wu, Xiangchao Ding, Cheng Zhou, Ping Ye, Yuan Sun, Jie Wu, Anchen Zhang, Xiaofan Huang, Lingyun Ren, Ke Wang, Peng Deng, Zhang Yue, Jiuling Chen, Sihua Wang, Jiahong Xia

**Affiliations:** 1 Department of Cardiovascular Surgery, Union Hospital, Tongji Medical College, Huazhong University of Science and Technology, Wuhan, China; 2 Department of Cardiology, Central Hospital of Wuhan, Tongji Medical College, Huazhong University of Science and Technology, Wuhan, China; 3 Department of Anesthesiology, Central Hospital of Wuhan, Tongji Medical College, Huazhong University of Science and Technology, Wuhan, China; 4 Department of Respiratory and Critical Care Medicine, Tongji Hospital, Tongji Medical College, Huazhong University of Science and Technology, Wuhan, China; 5 Department of Cardiovascular Surgery, Tongji Hospital, Tongji Medical College, Huazhong University of Science and Technology, Wuhan, China; 6 Department of Thoracic Surgery, Union Hospital, Tongji Medical College, Huazhong University of Science and Technology, Wuhan, China; 7 Department of Cardiovascular Surgery, Central Hospital of Wuhan, Tongji Medical College, Huazhong University of Science and Technology, Wuhan, China

## Abstract

Graft arteriosclerosis (GA) is the leading cause of late cardiac allograft dysfunction. The innate immune system plays a major role in GA, paprticularly Toll-like receptor 4 (TLR4) signaling. Here we characterized the role of TLR4 and its antagonist TAK-242 in a mouse model of GA. BALB/c (H-2d) donor aortas were transplanted into C57BL/6 (H-2b) recipients, and the mice received intraperitoneal injection of 3 or 10 mg/kg of TAK-242 or vehicle every other day for 1, 2, 4, 6, 8 and 12 weeks. With TAK-242 administration, intimal hyperplasia initially appeared at 2 weeks after transplantation, and TAK-242 postponed the progression of neointimal formation in allogeneic aortic grafts. TAK-242 treatment reduced CD68+ macrophage accumulation in the allografts, reduced the levels of ly-6C^hi^ monocytes in peripheral blood, bone marrow and spleen, and downregulated proinflammatory cytokine and chemokine levels. *Ex vivo* we observed that TAK-242 could improve the graft microenvironment by interfering the Tck/Mφ IL12p70 and IFNγ axis, reducing CCL2-mediated migration of vascular smooth cells.

## Introduction

Graft arteriosclerosis (GA) of transplanted organs is characterized by diffuse intimal hyperplastic lesions and results from compromised blood flow and ischemia^1^. Despite recent progress in the development of T cell-based immunotherapies for acute allograft rejection, GA remains the leading cause of late allograft dysfunction.

Transplanted grafts may encounter multipe adverse events, from ischemia-reperfusion injury, acute rejection episodes and chronic allograft inflammation to fibrosis, all contributing to GA. More recently, increasing evidence indicates that cells and proteins of the innate immune system play a major role in allograft damage^2,3^. In the early post-transplant stage, the inflammatory milieu shapes T-cell differentiation^4^, and innate recognition of the graft and its activation promotes rejection^5^. Accordingly, GA injury may be attenuated through therapeutic targeting of innate immune system receptors, signaling molecules, transcription factors or inflammatory mediators. Amongst these targets, Toll-like receptor 4 (TLR4) is responsible for innate immune activation^6^, and has been implicated in the pathogenesis of ischemia-reperfusion injury^7,8^.

TAK-242 (resatorvid), a small-molecule specific inhibitor of TLR4 signaling, inhibits the production of lipopolysaccharide (LPS)-induced inflammatory mediators by binding to the intracellular domain TIRAP of TLR4^9^. As a result, it completely prevents NF-κB DNA binding, and accordingly represses expression of IL-1β, IL-6, TNF-α, MIP-2 and CCL2^10^. In phase III clinical trials, TAK-242 has demonstrated promising efficacy as an antisepsis agent^11,12^. And recent evidence demonstrated that TAK-242 both effectively reducing inflammatory injury and neurological deficits in a mouse model of intracerebral hemorrhage^13^ or attenuated expression of IL-17A thereby improving LPS-induced lung inflammation^14^.

In this study, we evaluated the role of TLR4 and its antagonist TAK-242 in the development of GA in a murine allogeneic aorta transplantation model, and explore the underlying mechanisms.

## Results

### TAK-242 administration ameliorates intimal hyperplasia and postpones neointimal formation

**Figure 1:**
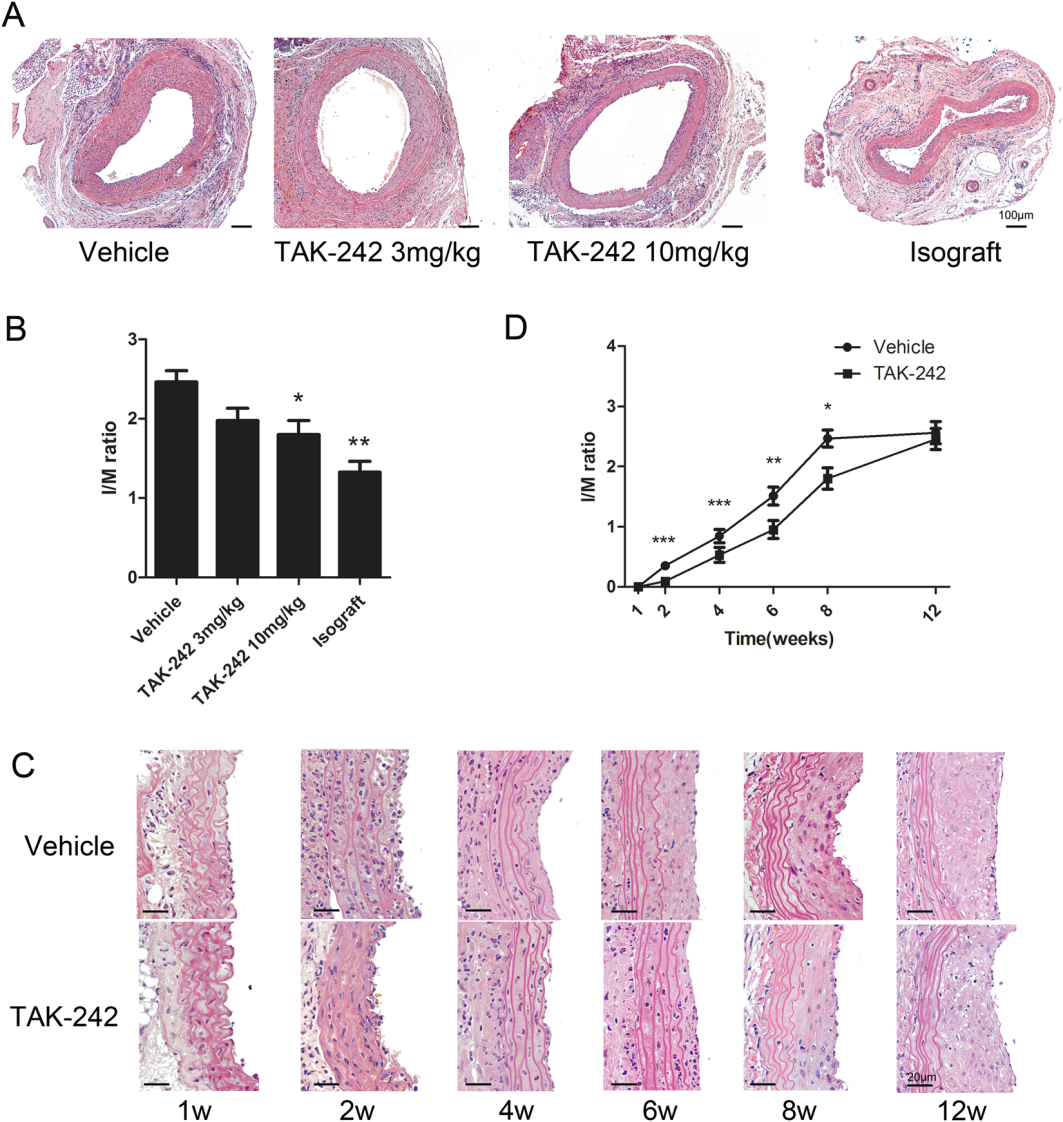
TAK-242 administration postpones neointimal formation. The intimal hyperplasia lesions in allografts at week 8 were measured (**A**), and the severity of intimal hyperplasia was quantified as I/M ratio, scale bar = 100 μm (**B**). The intimal hyperplasia lesions in Vehicle- and TAK-242-treated allografts at week 1, 2, 4, 6, 8 and 12 were measured (**C**), and the severity of intimal hyperplasia was quantified as I/M ratio, scale bar = 20 μm (**D**). (n = 8–9 mice for per group, 5 cross-sections for per graft, *P < 0.05, **P < 0.05, ***P < 0.001).

While isografts appeared healthy 8 weeks after transplantation, allografts exhibited intimal hyperplasia lesions at 8 weeks after transplantation (Fig. 1A). At 8 weeks, grafts treated with 3 mg/kg or 10 mg/kg TAK-242 every other day exhibited attenuated concentric intimal hyperplasia in comparison to vehicle-treated allografts (P = 0.0593, P = 0.0266, respectively, Fig. 1B). To determine whether TAK-242 ameliorates intimal hyperplasia in allogeneic aorta transplantation, 10 mg/kg TAK-242 was administrated and grafts were harvested at weeks 1, 2, 4, 6, 8 and 12 after transplantation. Intimal hyperplasia initially appeared at 2 weeks after transplantation in allogeneic aortic grafts (P < 0.001, Fig. 1C). In comparison to the vehicle-treated group, treatment with 10 mg/kg TAK-242 every other day significantly attenuated the concentric intimal hyperplasia at week 4, 6 and 8 (P < 0.001, P = 0.0019, P = 0.0265, respectively), while no significant difference was observed at week 12, suggesting that TAK-242 could postpone progression of neointimal formation (P = 0.4148, Fig. 1D).

**Figure 2:**
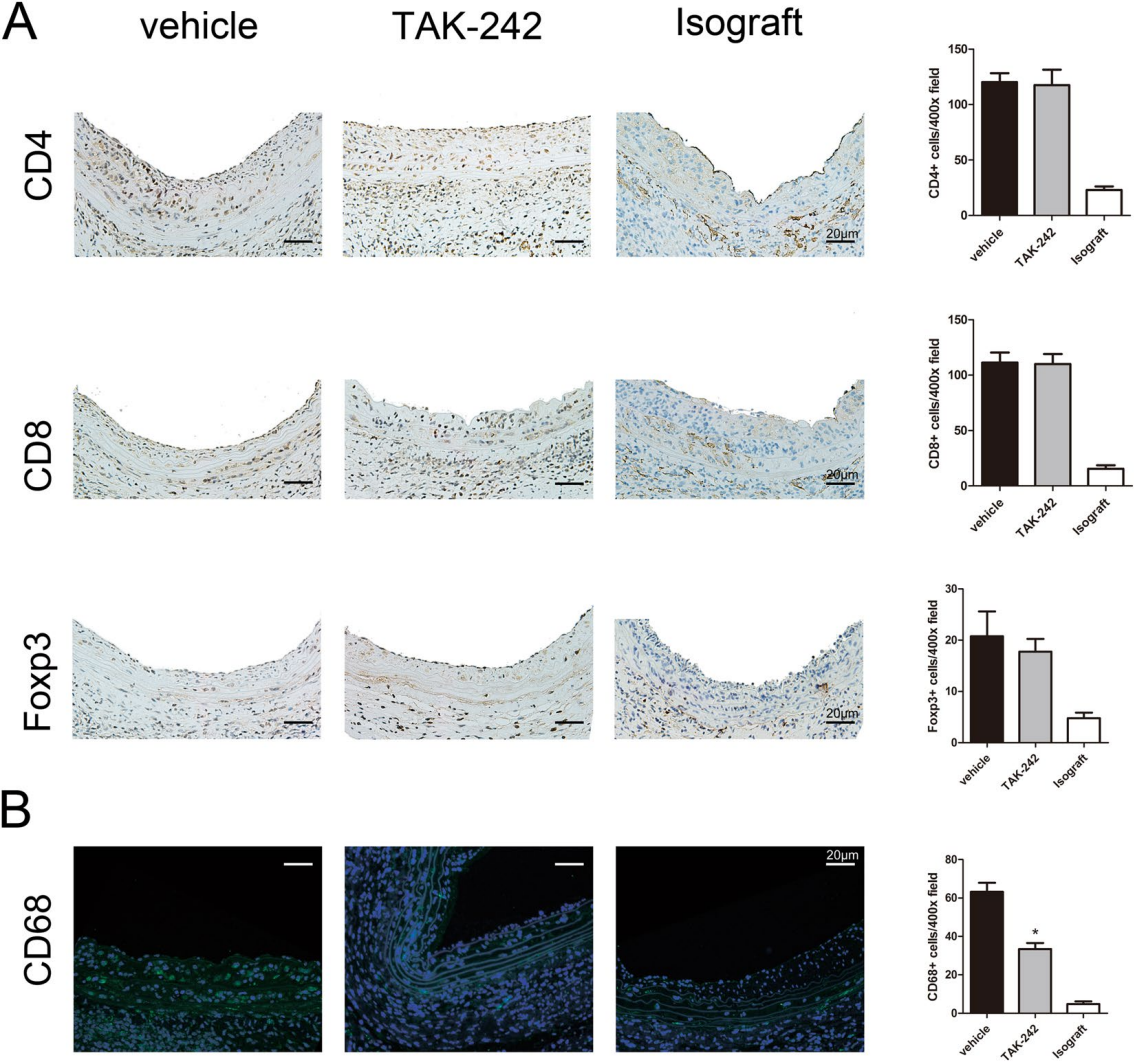
TAK-242 administration results in reduced CD68+ macrophage accumulation at week 2. (**A**) Immunohistochemical analysis showed no significant differences in the number of CD4+ , CD8+ or Foxp3+ T cells between the TAK-242-treated group and vehicle-treated group. (**B**) Immunofluorescence staining showed a reduction in CD68+ macrophages in TAK-242-treated allografts as compared with vehicle-treated allografts. (n = 8–9 mice for per group, 5 cross-sections for per graft, *P < 0.05, scale bar = 20 μm).

### TAK-242 administration reduces CD68+ macrophage accumulation

To further investigate the mechanism by which TAK-242 could inhibit arterial allograft intimal hyperplasia from week 2 after transplantation, immunohistochemical or immunofluorescence staining for CD4+ and CD8+ T cells, Foxp3+ regulatory T (Treg), and CD68+ macrophage accumulation was performed in vehicle-treated allografts, TAK-242-treated allografts and isografts at week 2 after transplantation (Fig. 2A,B). Infiltration of CD4, CD8, Foxp3+ Tregs and CD 68 cells was greater in both vehicle-treated allografts and TAK-242-treated allografts than isografts. Although both allograft groups showed comparable accumulation of CD4+ , CD8+ T cells and Foxp3+ Tregs, TAK-242-treated allografts demonstrated significantly fewer CD68+ macrophages in comparison to vehicle-treated allografts (Fig. 2B, 33.50 ± 3.122 *vs*. 63.25 ± 4.697 per field, P = 0.0019).

### TAK-242 administration reduced levels of Ly6C^hi^ cells in peripheral blood, bone marrow and spleen

**Figure 3:**
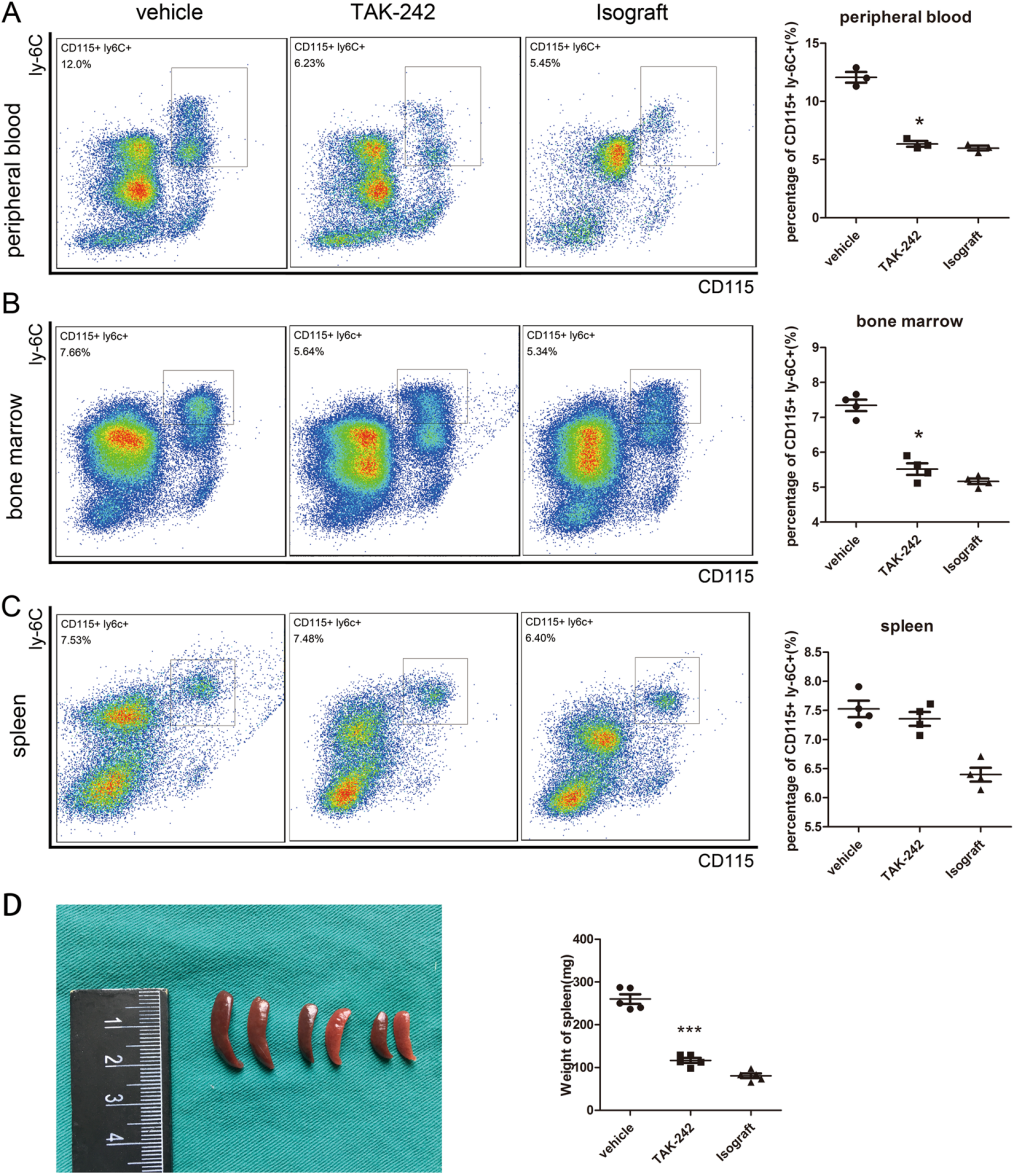
TAK-242 administration results in reduced Ly6C^hi^ cells in peripheral blood, bone marrow and spleen. Flow cytometry analysis showed that TAK-242 treatment could reduce the percentage of ly-6C^hi^ monocytes among CD11b+ cells in peripheral blood (**A**) and bone marrow (**B**), but not the spleen (**C**) after allogeneic aorta transplantation, as compared with vehicle-treated control. (**D**) Spleens were obtained from the vehicle-, TAK-242-treated allogeneic or isogeneic transplanted mice. TAK-242 treatment resulted in reduced weight of spleen. (n = 4 mice for per group, *P < 0.05; **P < 0.01).

Infiltrating macrophages are mostly derived from circulating monocytes which are recruited to the arterial intima^15^. To investigate how TAK-242 affects the recruitment of circulating monocytes, peripheral blood, bone marrow and spleen monocytes from allogeneic transplanted mice were assessed by flow cytometry. Peripheral blood Ly6C^hi^ monocytes accounted for 12.07 ± 0.47% of CD11b+ cells in vehicle-treated mice, but 6.35 ± 0.24% in TAK-242-treated mice (P < 0.001, Fig. 3A). Similarly, bone marrow Ly6C^hi^ monocytes accounted for 7.35 ± 0.16% and 5.52 ± 0.17 of CD11b+ cells in vehicle-treated and TAK-242-treated mice, respectively (P < 0.001, Fig. 3B). Meanwhile, the spleens of vehicle-treated mice weighed 260.0 ± 11.10 mg, and contained 7.53 ± 0.14% Ly6C^hi^ monocytes, while the spleens of TAK-242-treated mice weighed 116.7 ± 5.80 mg, and contained 7.36 ± 0.12% Ly6C^hi^ monocytes (P = 0.3921, Fig. 3C).

### TAK-242 administration downregulates proinflammatory cytokines and chemokines

The expression of genes encoding cytokines promoting lymphocyte differentiation and growth (IFN-γ, IL-12, IL-2, IL-4), inflammation (IL-6, TNFα, IL-1β), cytotoxicity (iNOS), and immune modulation (IL-10, TGF-β), and chemokines (CCL2, CCL5, CXCL10) was assessed 2 weeks after transplantation. Expression of all studied genes was found to be significantly higher in the allografts than isografts. Compared with vehicle-treated allografts, TAK-242-treated allografts exhibited significantly reduced expression of IL-12 (P = 0.491), IL-6 (P = 0.012), TNFα (P = 0.016), IL-1β (P = 0.043), iNOS (P = 0.005), and CCL2 (P < 0.001), but significantly increased expression of IL-4 (P < 0.001). There were no significant differences in the expression of other genes (IFN-γ, IL-2, CCL5, CXCL10, IL-10, TGF-β) between TAK-242-treated allografts and vehicle-treated allografts.

### TAK-242 administration negatively regulates the NF-κB signaling pathway in macrophages

**Figure 4:**
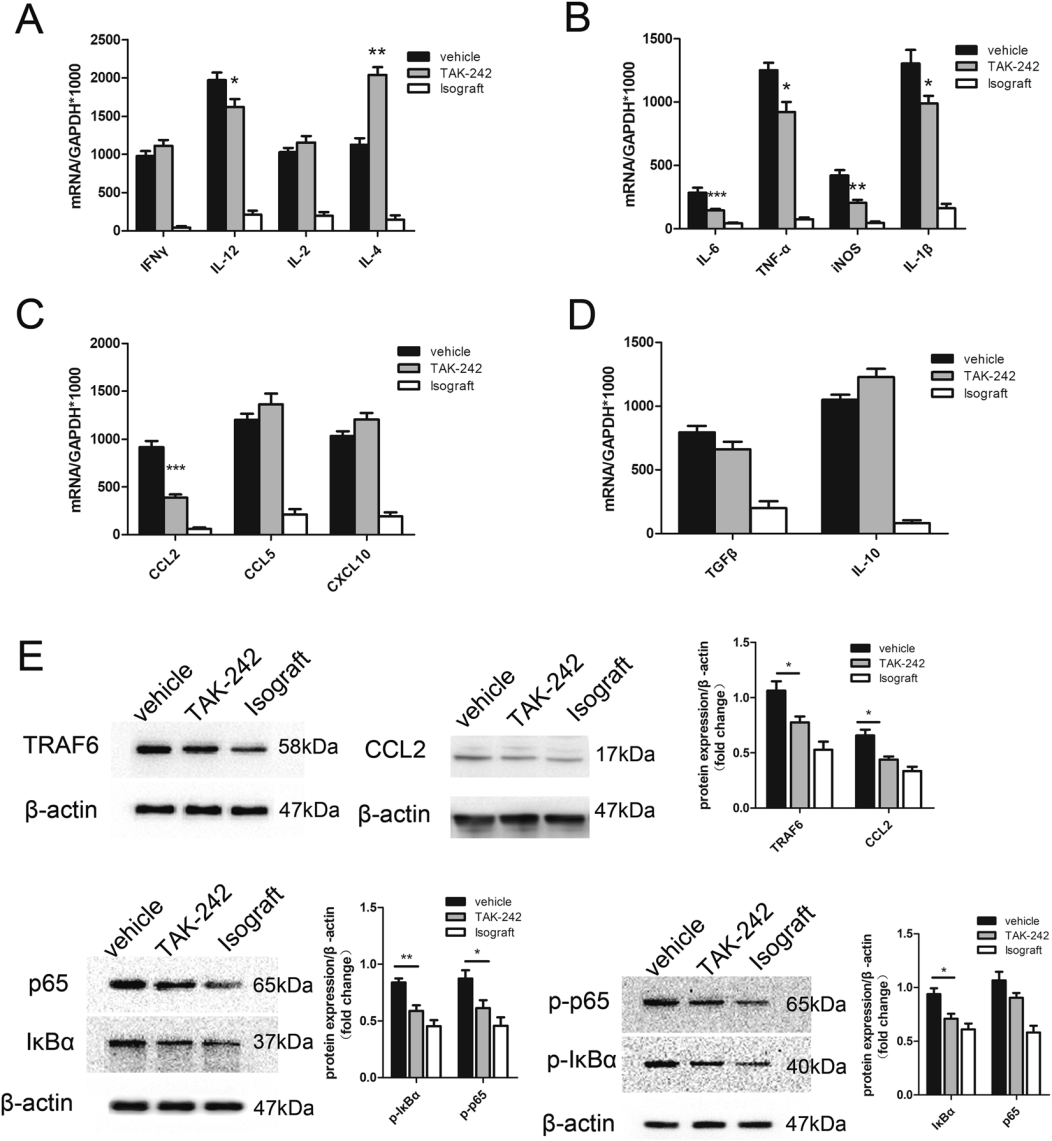
TAK-242 downregulates proinflammatory cytokines in grafts and NF-κB signaling pathway in graft macrophages. TAK-242 treatment significantly reduced expression of (**A**) IL-12 (P = 0.0491), (**B**) IL-6 (P = 0.012), TNFα (P = 0.016), iNOS (P = 0.005), IL-1β (P = 0.043), and (**C**) CCL2 (P < 0.001) and increased expression of (A) IL-4 (P < 0.001) in allografts as compared with vehicle treatment. There was no significant difference in the expression of other genes (**A**,**B**,**C**,**D**) between the two groups. (**E**) TAK-242 treatment significantly decreased TRAF6 (P = 0.036) and CCL2 (P = 0.023) expression and decreased phosphorylation of IκBα (P = 0.010) and p65 (P = 0.049) in graft macrophages, as compared with vehicle treatment. (n = 4 mice for per group *P < 0.05, **P < 0.05, ***P < 0.001).

It has been reported that TLR4-mediated NF-κB signaling is critical for the production of TNFα- IL-6 and IL-12^8^. To evaluate alterations in macrophage function in the presence or absence of TAK-242, we investigated the activation of key molecules of the NF-κB signaling pathway in macrophages isolated from allografts and isografts. We found that TAK-242 administration significantly decreased expression of TRAF6, which was accompanied with synchronously decreased phosphorylation of IκBα and p65, in comparison to vehicle treatment (Fig. 4E). These data indicated that treatment with TAK-242 inhibited activation of the NF-κB signaling pathway in macrophages of allografts. Thus, TAK-242 affects not only generation of, but also the function of graft macrophages.

### TAK-242 administration impairs the IL12p70 and IFNγ axis

To simulate the process *in vivo*, a Tck/Mφ co-culture system was used^16^. Either cytokine-stimulated T cells (Tck) or Toll-like receptor (TLR) ligand induced the production of TNF-α and IL-6. Synergistic release of TNFα and IL-6 was observed when macrophages were exposed to a TLR4 ligand together with Tck. However, TAK-242 significantly reduced production of TNFα or IL-6 in the presence of TLR4 ligand and Tck.

**Figure 5:**
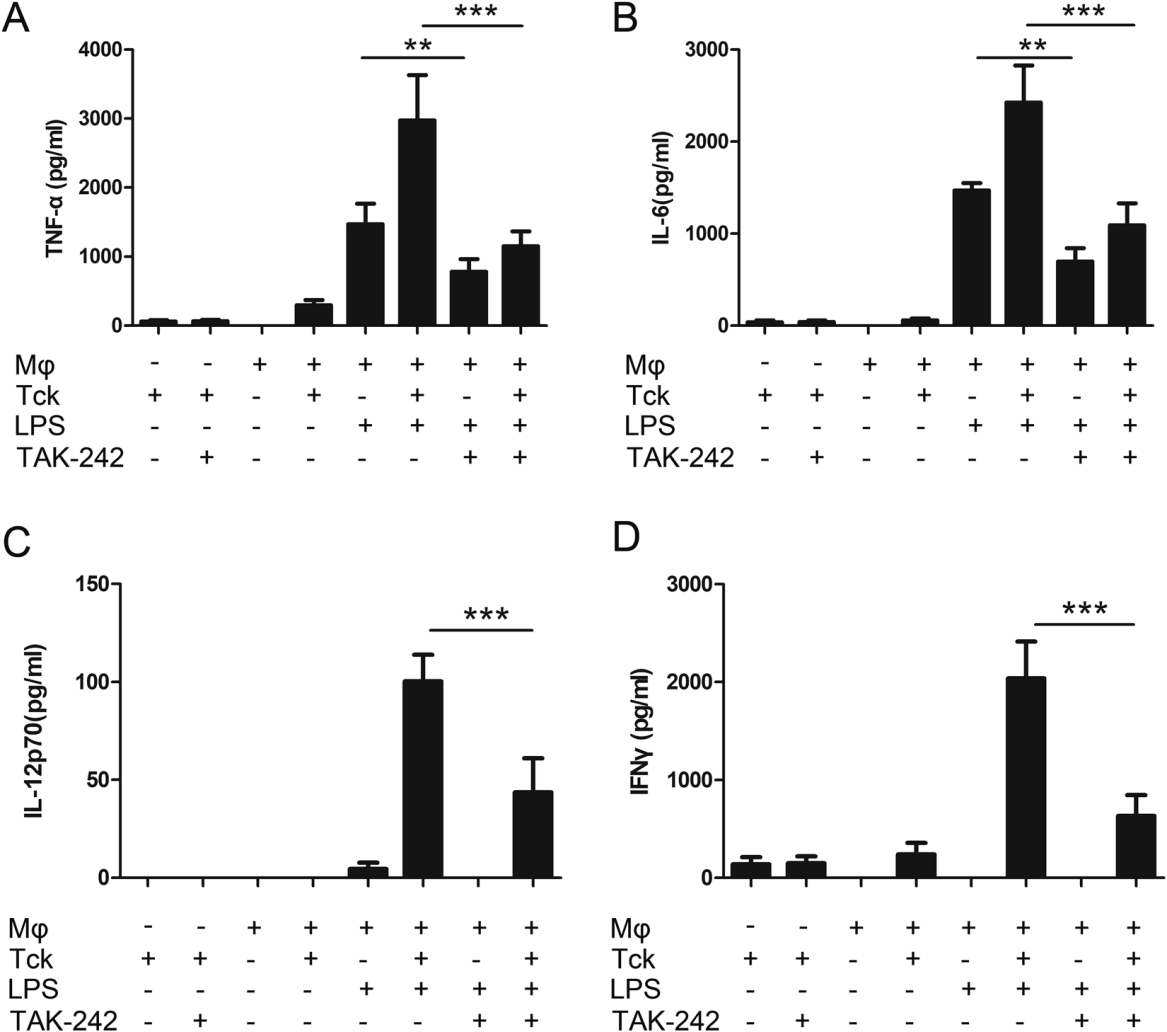
TAK-242 administration impairs the IL12p70 and IFNγ axis in Tck/Mφ co-culture system. Either cytokine-stimulated T cells (Tck) or Toll-like receptor (TLR) ligand induced the production of TNF-α (**A**) and IL-6 (**B**). Exposure to TLR4 ligand together with Tck led to synergistic release of TNFα and IL-6, which could be abrogated by TAK-242 treatment. Stimulation of macrophages with TLR ligand induced the production of marginal level of IL-12p70 and no IFNγ, while stimulation of macrophages with Tck alone induced the production of a small amount of IFNγ and no IL-12p70 (**C** and **D**); stimulation with TLR4 ligands in combination with Tck resulted in a remarkable release of IL-12p70 and IFNγ. Blockade of TLR4 by TAK-242 contributed to a significant reduction in the production of IL-12p70 and IFNγ. (n = 5 independent experiments, *P < 0.05, **P < 0.05, ***P < 0.001).

The IL-12p70/IFNγ axis appears to play an important role in the pathogenesis of GA^17^. Therefore, we also investigated whether TAK-242 might additionally modulate this pathway. Stimulation of macrophages with TLR ligand induced the production of marginal level of IL-12p70 and no IFNγ, while stimulation of macrophages with Tck alone induced the production of a small amount of IFNγ and no IL-12p70; However, stimulation with TLR4 ligand in combination with Tck resulted in a remarkable release of IL-12p70 and IFNγ (Fig. 5C and D). In contrast, blockade of TLR4 by TAK-242 result significantly reduced IL-12p70 and IFNγ production (Fig. 5C,D).

### TAK-242 administration reduces CCL2-mediated migration of VSMCs towards CCL2

**Figure 6:**
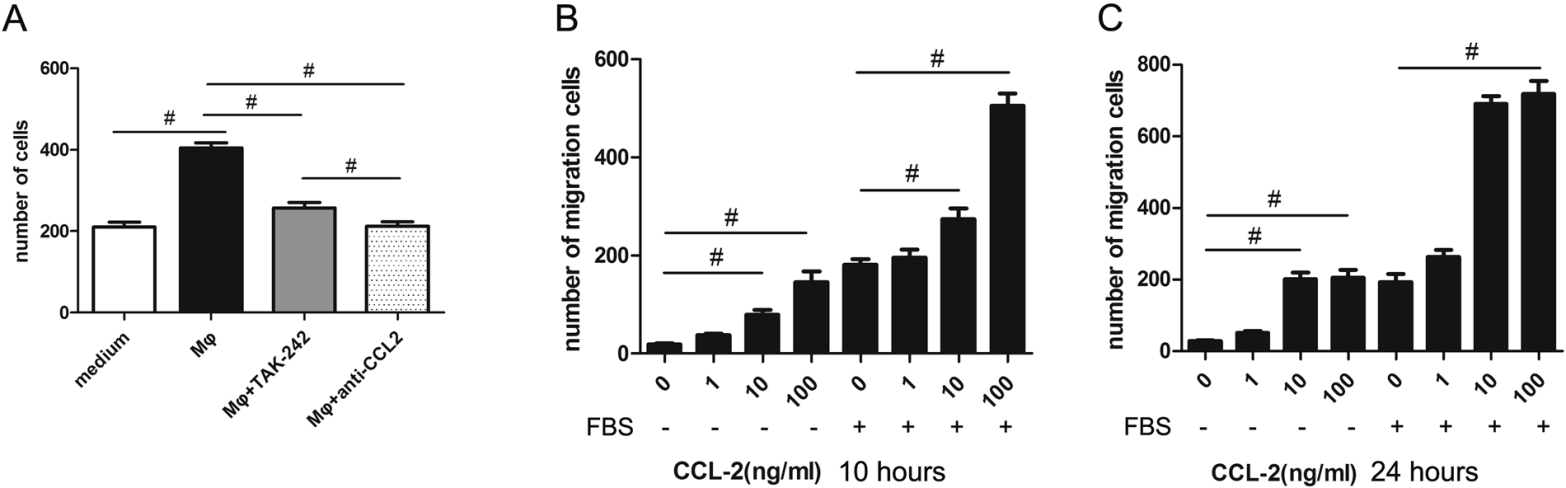
TAK-242 reduces the migration of VSMCs towards CCL2. (**A**) VSMCs migration was induced by macrophages and neutralized by anti-CCL2 antibody. The VSMC migration towards CCL2 was examined by transwell assay at 10 (**B)** and 24 hours (**C**) after seeding. At 10 hours, CCL2 induced VSMC migration in a dose-dependent manner, irrespective of the presence of FBS. At 24 hours, 10 ng/ml CCL2 significantly promoted VSMC migration. There was no significant difference in the number of migrated VSMCs between the high (100 ng/ml) and low (10 ng/ml) concentrations of CCL2. (n = 5 independent experiments, # Bonferroni corrected level of significance <0.05).

The migration capacity of VSMCs towards CCL2 in DMEM supplemented with or without 10% FBS was assessed in transwell assay at 10 hours and 24 hours (Fig. 6B). At 10 hours after cell seeding, CCL2 induced VSMC migration in a dose-dependent manner irrespective of supplementation with FBS. However, FBS further promoted migration of VSMCs. At 24 hours, 10 ng/ml CCL2 significantly promoted migration of VSMCs. There was no significant difference in VSMC migration between the high (100 ng/ml) and low (10 ng/ml) concentrations of CCL2 (Fig. 6C).

The migration of VSMCs could be significantly induced by macrophages and neutralized by anti-CCL2 antibody (Fig. 6A). Interestingly, administration of TAK-242 contributed to a significant reduction in the number of migrated VSMCs.

## Discussion

In this study, we investigated the effects of TLR4 antagonist TAK-242 on development of graft atherosclerosis in a model of aorta transplantation. At week 2 posttransplantation, TAK-242 treatment reduced aortal intima thickness; however, at day 84, similar I/M ratios were observed between vehicle- and TAK-242-treated mice. In further histological examination performed at week 2, TAK-242 treatment significantly enhanced IL-4 expression, and decreased expression of IL-12, IL-6, TNF-α, iNOS, CCL2 and the accumulation of CD68+ macrophages in allografts, compared with vehicle-treated mice. Moreover, the proportion of ly6C^hi^ monocytes in peripheral blood and bone marrow and spleen was decreased in TAK-242 treated mice. And western blotting demonstrated that TAK-242 negatively regulated the NF-κB signaling pathway in graft macrophages. In a Tck/Mφ co-culture system designed to simulate the circumstance *in vivo*, TAK-242 administration reduced TNF-α and IL-6 production, and impaired the IL12p70 and IFNγ axis. In addition, TAK-242 administration reduced migration of VSMCs toward chemokine CCL2 *ex vivo*.

Toll-like receptor signaling may contribute to progression of graft atherosclerosis. TLR4 is highly expressed on monocytes and macrophages. In the early stage after transplantation, ischemic tissue damage occurs during the process of organ removal, preservation and implantation, leading to the release of endogenous TLR ligands, such as HMGB1, heat shock protein 70 (Hsp 70) and subsequent innate inflammatory responses^18,19^. The results presented here are consistent with our previous findings that the blockage of TLR4 signaling by neutralizing HMGB1 was associated with modestly better graft function, and delayed rejection post ischemia-reperfusion injury in a cardiac transplantation model^20^.

The second way by which TLR4 can promote graft damage is through upregulation of pro-inflammatory mediators including cytokines and chemokines, such as CD80 and CD40, thereby potentiating the development of adaptive alloimmunity^21^. It’s known that TLR4, stimulated by LPS and other ligands, drives macrophages to a preferentially M1 phenotype. Characterized by high antigen presentation, high production of IL-12 and IL-23 and high production of nitric oxide (NO), inflammatory M1 macrophages produce many other pro-inflammatory cytokines like TNFα, IL-1β, IL-6, IL-12, CXCL1-3, CXCL-5, and CXCL8-10^22,23^. In the present study, TAK-242 treatment significantly reduced expression of IL-6, TNF-α, IL-1β, and CCL2, in accordance with a previous work in cigarette smoke-induced pulmonary^24^. Although the expression of costimulatory markers on antigen-presenting cells was not assessed, it remains possible that antigen presentation in TAK-242-treated grafts was impaired, thus delaying alloimmune-mediated graft injury in our model.

Another intriguing possibility is that the blockage of TLR4 signaling may interfere the IL12p70 and IFNγ axis. IFNγ is included in combination with TLR4 signaling in the M1/M2 paradigm, and gene expression profiles of the combination are different from TLR4 signaling or IFNγ profiles alone^25,26^. As reported, infiltration of macrophages in allografts exacerbates tissue damage during acute rejection episodes; and chronically, macrophages contribute to chronic allograft inflammation and fibrosis^27,28^. In addition to mediating innate immune responses, macrophages serve as amplifiers of the adaptive immune response by supporting T-cell reactions within allografts^29^. In our model, TAK-242 treatment significantly reduced production of IL-12p70, IFNγ and CCL2. Thus, TAK-242 impedes the capacity of macrophages to create a permissive microenvironment for the development of graft atherosclerosis.

Our current findings indicate that TAK-242 monotherapy could disrupt TLR4 signaling to ameliorate intimal hyperplasia at week 2, but the effect was not sufficient to prevent graft atherosclerosis. Previous studies reported that deficient of MyD88 or short-term MyD88 inhibition promoted the survival of grafts^30,31^. Downstream of TLR4, two adaptors, MyD88 and TRIF, mediate the signaling. The signaling pathway through the MyD88 adaptor results in the activation of a cascade of kinases, including IRAK4, TRAF6, and IKKβ, which finally leads to the activation of nuclear factor kappa B (NF-κB). As a key transcription factor related to macrophage M1 activation, NF-κB regulates the expression of a large number of inflammatory genes including TNFα, IL-1β, cyclooxygenase 2 (COX2), IL-6, and IL12p40. The present findings were consistent with those reports. However, TAK-242 is a highly selective inhibitor of TLR4, and may represent a safer approach for TLR4-related diseases^32^. In our previous studies, we demonstrated that smooth muscle-like cells might be the dominant cell type at the late stage of graft atherosclerosis following ischemia-reperfusion injury^33,34^. The autonomous loop of interferon-γ production by smooth muscle cells may take the place of the Tck/Mφ IL12p70 and IFNγ axis^17^.

In conclusion, inhibition of TLR4 with its antagonist TAK-242 attenuates intimal hyperplasia in the short-term by downregulating the function of macrophages and interfering with the IL12p70 and IFNγ axis. Therefore, TLR4 antagonists may have therapeutic potential in prevention of pathological progression of GA.

## Methods and Methods

### Mice

The study was approved by the Institutional Animal Care and Use Committee at Tongji Medical College, Huazhong University of Science and Technology (Wuhan, China). The methods were carried out in accordance with the relevant guidelines and regulations of the Institutional Animal Care and Use Committee at Tongji Medical College, Huazhong University of Science and Technology. BALB/c (B/c; H-2d) and C57BL/6 (H-2b) mice were purchased from HFK Bioscience Co., Ltd. (Beijing, China). All murine studies were performed in the Specific-Pathogen-Free Laboratory Animal Facility of Tongji Medical College.

### Aorta transplantation

All mice were male, aged 6- to 8-weeks. For isotransplantation, B6 were used as both donors and recipients; while in allotransplantation, Balb/c mice were transplanted into B6. Aortic transplantation surgery was performed as previously reported^33^. For the treatment group, TAK-242 (3 mg/kg or 10 mg/kg) in 1% Dimethyl sulfoxide (DMSO) was administered by intraperitoneal injection every other day from the day before surgery to day 84 after surgery. Control mice received an equivalent volume of 1% DMSO. The grafts were harvested at week 1, 2, 4, 6, 8 and 12 after surgery and were then processed for histological analysis.

### Tissue staining and morphometric analysis

Paraffin sections of the harvested grafts were cut at 5-μm thickness. H&E staining was performed using routine techniques.

Sections for immunofluorescence were incubated with anti-CD68 (Abcam, Cambridge, UK; ab125212) overnight, followed by incubation with fluorescently labeled secondary antibodies (AntGene Biotech, Wuhan, China). Nuclei were stained using 4.6-diamidino-2-phenylindole (DAPI; Linaris, Wertheim, Germany). Isotype-matched control antibodies (Abcam, ab172730) served as negative controls.

Immunohistochemistry was performed as described previously^35^. Primary antibodies included anti-CD4 antibody (Abcam, ab183685), anti-CD8 antibody (Abcam, ab203035) and anti-Foxp3 antibody (Abcam, ab54501). For negative controls, primary antibodies were omitted.

The severity of allograft vasculopathy was assessed on elastin-stained arteries, using the neointimal (I)/medial (M) ratio^33^. The total number of CD4+ , CD8+ , Foxp3+ and CD68+ cells in each section was counted manually by technicians blinded to the sample origin. Three sections from each group were selected for analysis using the IMAGE-PRO PLUS 6.0 software (Media Cybernetics, Inc., Silver Spring, MD). Error bars represent the standard errors of mean (SEM).

### Real-time polymerase chain reaction (PCR)

Total RNA was isolated from tissue or harvested cells using TRIzol® reagent (Invitrogen). cDNA synthesis kit (Takara, D6110A) was used to generate complementary DNA mixture. The sequences of the primers used are shown in Table 1. Quantitative PCR was performed on an ABI StepOne Plus System (Applied Biosystems, Foster City, CA) using a SYBR Premix Ex Taq (Takara, Otsu, Shiga, Japan) following the manufacturer’s protocol. mRNA levels were normalized to GAPDH using the 2^−ΔΔCT^ method^36^.

**Table 1:** The sequences of the primers used.

	Forward (5′–3′)	Reverse (5′–3′)
IFN-γ	GAACTGGCAAAAGGATGGTGA	TGTGGGTTGTTGACCTCAAAC
IL-12p40	GACCATCACTGTCAAAGAGTTTCTAGAT	AGGAAAGTCTTGTTTTTGAAATTTTTTAA
IL1β	GAAATGCCACCTTTTGACAGTG	TGGATGCTCTCATCAGGACAG
IL-2	TGCTCCTTGTCAACAGCG	TCATCATCGAATTGGCACTC
IL-4	ATGGGTCTCAACCCCCAGCTAGT	GCTCTTTAGGCTTTCCAGGAAGTC
IL-6	GAGGATACCACTCCCAACAGACC	AAGTGCATCATCGTTGTTCATACA
TNF-α	CATCTTCTCAAAATTCGAGTGACAA	TGGGAGTAGACAAGGTACAACCC
iNOS	CCTTGTTCAGCTACGCCTTC	GGTATGCCCGAGTTCTTTCA
CCL2	CCCAATGAGTAGGCTGGAGA	GCTGAAGACCTTAGGGCAGA
CCL5	ATATGGCTCGGACACCACTC	GTGACAAACACGACTGCAAGA
CXCL10	CCACGTGTTGAGATCATTGC	AGTAGCAGCTGATGTGACC
TGFβ	ACAGGGCTTTCGATTCAGCG	CACTTGCAGGAGCGCACAAT
IL-10	TGCCTTCAGCCAGGTGAAGACT	AAATTCATTCATGGCCTTGTA
GAPDH	TTCACCACCATGGAGAAGGCCG	GGCATGGACTGTGGTCATGA

### Flow cytometry

Animals were anesthetized at week 2 after transplantation by intraperitoneal injection of phenobarbital (10 mg/kg), then 100 μl peripheral blood, bone marrow and 1 mg spleen tissues were harvested. Single cell suspensions were generated from bone marrow and spleen tissues by mechanical disruption using a gentleMACS Dissociator (Miltenyi Biotec, Bergisch Gladbach, Germany) for 1 min. Blood, bone marrow and spleen cell suspensions were then suspended in 0.83% ammonium chloride solution containing 10% (v/v) Tris buffer (pH 7.65) to lyse erythrocytes. The cell suspension was centrifuged at 400 × g for 10 min at 4 °C and purified by density centrifugation. Cell preparations were stained using the following fluorochrome-conjugated antibodies: CD11b-FITC, CD115-PE, Ly6C-PERCP-Cy5.5 (BD Biosciences, San Jose, CA, USA).

### Western blot analysis

The grafts harvested from mice that received aortic transplants were cut into small pieces and dissociated into single cell suspension at 2 weeks. Cells were incubated in DMEM medium containing 0.2% collagenase type II (Worthington Biochemical) at 37 °C for 1 hour. Cells were filtered through a 30-μm nylon sieve (BD) before using CD11b microbeads and AutoMACS (Miltenyi Biotec, 130-049-601). The macrophages were lysed with sodium dodecyl sulfate (SDS) sample loading buffer. Lysates were boiled in sample buffer (200 mmol/l Tris [pH 6.8], 20% glycerol, 2% SDS, 0.1% bromophenol blue, and 10% β-ME). Nuclear and cytoplasmic protein samples were prepared using the nuclear and cytoplasmic extraction reagent kit (Beyotime, Shanghai, China) for the NF-κB translocation assay. Then the same amount of total protein from each lysate was loaded onto a 12% SDS-polyacrylamide gel electrophoresis (PAGE) gel. After electrophoresis, proteins were transferred onto a nylon polyvinylidene fluoride (PVDF) membrane which was blocked overnight with 4% nonfat dry milk in Tris-buffered Saline-Tween (100 mmol/l Tris, pH 7.5, 0.9% NaCl, 0.1% Tween-20). Thereafter, the membrane was incubated for 1 h at room temperature with Anti-TRAF6 (Abcam, ab33915), Anti-IκBα(ab7217), Anti-p-IκBα(ab133462), Anti-p-p65 (ab86299), Anti-p65 antibody (ab32536), anti-CCL2 (sc-1784, Santa Cruz, California, USA) or β-actin (GB13001-1, Goodbio, Wuhan, China). Membranes were washed and incubated for 1 h with horseradish peroxidase–labeled goat anti-rabbit IgGs (Cedarlane, Burlington, NC, USA). Blots were developed using enhanced chemiluminescence (ECL, GE Healthcare BioSciences, Pittsburgh, PA, USA).

### Cell culture

Bone marrow was collected from C57BL/6 mice and erythrolysed with 0.83% ammonium chloride solution containing 10% (v/v) Tris buffer (pH 7.65). Purified bone marrow cells were cultured in RPMI-1640 medium containing macrophage colony-stimulating factor (800 U/ml, Pepro Tech, Oak Park, CA) for 4 days. Non-adherent cells were removed by aspiration and by washing with RPMI 1640 medium and approximately 92% of the remaining macrophages were CD11b-positive cells (determined by flow cytometry^34^).

T cells were obtained from the spleen of the same donor using CD4 microbeads and an AutoMACS (Miltenyi Biotec, 130-104-454, Bergisch Gladbach, Germany). CD4+ T cells were cultured in a complete medium containing recombinant mouse IL-2 (25 ng/ml, P04351), IL-6 (100 ng/ml, P08505) and TNFα (25 ng/m, P06804) (R&D China, Shanghai, China) at a density of 2 × 10^6^/ml for 4 days, and then the cells are harvested.

Macrophages were harvested, washed, counted and then plated at a concentration of 5 × 10^4^ cells/well in 96-well culture plates at day 4. For cell contact assay, after extensive washing, 2 × 10^5^ cytokine-activated T cells were added to macrophages in the presence of LPS (1 ng/ml, Escherichia coli 055:B5; Solarbio, Beijing, China) or phosphate buffered solution (PBS). After 24 h, the supernatants were collected for analysis of cytokine levels. For TLR4 stimulation, TAK-242 was added to macrophages in the presence or absence of Tck, and cultured for 24 h before harvest.

Medial vascular smooth muscle cells (VSMCs) from the descending thoracic aortas were isolated and digested using type II collagenase as previously described^37^. Cells at passage 3 to 5 were used for the following experiments.

### Cell migration assay

Transwell assay was performed to evaluate VSMC migration in DMEM supplemented with 10% fetal bovine serum (FBS) in the presence or absence of macrophages in a transwell chamber (8-μm pore size; Corning). Briefly, macrophages were seeded into the lower chamber in DMEM medium containing 10% FBS and TAK-242, or CCL2 antibody (100 ng/ml, Santa Cruz, Santa Cruz, CA) that neutralized macrophage-induced migration. VSMCs (10^4^/well) were placed into the upper chamber in DMEM medium or DMEM containing 0.2% bovine serum albumin (BSA).

Recombinant Murine CCL2 (1–100 ng/ml, PeproTech 250–10, Rocky Hill, NJ) was added into the lower chamber. Cells on the filter were removed after 10 hours or 24 hours, and the cells in the bottom chamber were fixed and stained with DAPI. The number of cells that migrated across the filter was counted in five random fields (magnification × 100) using a fluorescence microscope.

### ELISA

Levels of IFNγ, TNFα, IL-12p70, IL-6 in culture supernatants were measured using commercially available kits (EMC101g.96, EMC102a.96, EMC006.96, and EMC004.96, respectively, NeoBioscience, Shenzhen, China) according to the manufacturer’s instructions. The absorbance was recorded using a microplate reader (Thermo Scientific) at 450 nm.

### Statistical analysis

Data were expressed as mean ± SEM. Unpaired Student’s t-test was used to evaluate the statistical differences, and P < 0.05 was considered to indicate significance. All experiments were repeated at least three times.
